# Population Coding of Facial Information in the Monkey Superior Colliculus and Pulvinar

**DOI:** 10.3389/fnins.2016.00583

**Published:** 2016-12-21

**Authors:** Minh N. Nguyen, Hiroshi Nishimaru, Jumpei Matsumoto, Quan Van Le, Etsuro Hori, Rafael S. Maior, Carlos Tomaz, Taketoshi Ono, Hisao Nishijo

**Affiliations:** ^1^System Emotional Science, Graduate School of Medicine and Pharmaceutical Sciences, University of ToyamaToyama, Japan; ^2^Primate Center and Laboratory of Neurosciences and Behavior, Department of Physiological Sciences, Institute of Biology, University of BrasíliaBrasilia, Brazil; ^3^Psychiatry Section, Department of Clinical Neuroscience, Karolinska Institute, Karolinska HospitalStockholm, Sweden

**Keywords:** superior colliculus, pulvinar, subcortical pathway, face, monkey

## Abstract

The superior colliculus (SC) and pulvinar are thought to function as a subcortical visual pathway that bypasses the striate cortex and detects fundamental facial information. We previously investigated neuronal responses in the SC and pulvinar of monkeys during a delayed nonmatching-to-sample task, in which the monkeys were required to discriminate among 35 facial photos of five models and other categories of visual stimuli, and reported that population coding by multiple SC and pulvinar neurons well discriminated facial photos from other categories of stimuli (Nguyen et al., 2013, 2014). However, it remains unknown whether population coding could represent multiple types of facial information including facial identity, gender, facial orientation, and gaze direction. In the present study, to investigate population coding of multiple types of facial information by the SC and pulvinar neurons, we reanalyzed the same neuronal responses in the SC and pulvinar; the responses of 112 neurons in the SC and 68 neurons in the pulvinar in serial 50-ms epochs after stimulus onset were reanalyzed with multidimensional scaling (MDS). The results indicated that population coding by neurons in both the SC and pulvinar classified some aspects of facial information, such as face orientation, gender, and identity, of the facial photos in the second epoch (50–100 ms after stimulus onset). The Euclidean distances between all the pairs of stimuli in the MDS spaces in the SC were significantly correlated with those in the pulvinar, which suggested that the SC and pulvinar function as a unit. However, in contrast with the known population coding of face neurons in the temporal cortex, the facial information coding in the SC and pulvinar was coarse and insufficient. In these subcortical areas, identity discrimination was face orientation-dependent and the left and right profiles were not discriminated. Furthermore, gaze direction information was not extracted in the SC and pulvinar. These results suggest that the SC and pulvinar, which comprise the subcortical visual pathway, send coarse and rapid information on faces to the cortical system in a bottom-up process.

## Introduction

The superior colliculus (SC) is a multilayered structure in the mammalian midbrain. Its superficial layers receive visual inputs from the retina (Perry and Cowey, 1984; Rodieck and Watanabe, 1993). The pulvinar, which is located in the posterior region of the thalamus, is proportionally larger in higher mammals, such as primates, and largest in the human brain (Browne and Simmons, 1984). The pulvinar receives visual inputs from subcortical structures, including the superficial and deep layers of the SC, and it has intimate reciprocal connections with a wide variety of cortical areas (Linke et al., 1999; Grieve et al., 2000; Kaas and Lyon, 2007). These neuroanatomical studies suggest that the SC and pulvinar form a subcortical visual route to the cortex that bypasses the striate cortex (Day-Brown et al., 2010; Pessoa and Adolphs, 2010; Tamietto and de Gelder, 2010; Tamietto et al., 2012; Rafal et al., 2015). Indeed, human subjects and monkeys with lesions in the striate cortex (V1) display a wide range of residual visual functions (i.e., blindsight) (Stoerig and Cowey, 1997). For example, monkeys and humans with striate cortical lesions can discriminate figures (Schilder et al., 1972) and forms (Perenin and Rossetti, 1996).

The SC and pulvinar project to other subcortical areas, including the amygdala and striatum (Day-Brown et al., 2010; Tamietto and de Gelder, 2010; Rafal et al., 2015). These subcortical routes might also be involved in the rapid processing of facial expression information (Morris et al., 2001; Tamietto and de Gelder, 2010), and the facial detection of infants with immature cortical visual systems might depend on the subcortical visual system (Johnson, 2005). Furthermore, human neuropsychological studies have reported substantial evidence that suggests that this subcortical pathway is involved in the discrimination of face gender and facial identity (Morris et al., 2001; Khalid et al., 2013; Gabay et al., 2014). Consistently, recent neurophysiological studies have reported that neurons in the monkey SC and pulvinar respond differentially to various photos of human and monkey faces, human facial expressions, and face-like patterns (Maior et al., 2010; Van Le et al., 2013; Nguyen et al., 2013, 2014). However, these SC and pulvinar neurons are broadly tuned, suggesting that single neurons may not code for gender or identity of the face. One way to simultaneously code for multiple types of facial information is through the population coding of broadly tuned neurons (Calder and Young, 2005; Meyers et al., 2015). Consistent with this idea, the previous studies investigated population coding of visual stimuli in the monkey SC and pulvinar with multidimensional scaling (MDS), and reported that population coding well discriminated facial photos from other categories of stimuli including simple geometrical figures, eye-like patterns and face-like patterns (Nguyen et al., 2013, 2014). However, it remains unknown whether population coding could represent multiple types of facial information including facial identity, gender, facial orientation, and gaze direction in the SC and pulvinar. In the present study, we reanalyzed the same neuronal responses in the monkey SC and pulvinar to human facial photos (Nguyen et al., 2013, 2014) with MDS in order to investigate the population coding of multiple types of facial information in the SC and pulvinar.

## Materials and methods

### Subjects and experimental setup

Two adult (one female and one male) macaque monkeys (*Macaca fuscata*) weighing 7.2–9.5 kg were used (Nguyen et al., 2013, 2014). The monkeys were treated in strict compliance with the United States Public Health Service Policy on the Humane Care and Use of Laboratory Animals, the National Institutes of Health Guide for the Care and Use of Laboratory Animals, and the Guidelines for the Care and Use of Laboratory Animals of the University of Toyama. Every effort was made to minimize the number of animals that were used and their suffering. The study was approved by the Committee for Animal Experiments and Ethics at the University of Toyama.

The monkey sat in a monkey chair that was 68 cm away from the center of a 19-inch computer display in a shielded room while performing the behavioral tasks during the training and recording sessions. The cathode ray tube monitor was set so that its center was on the same horizontal plane as the monkey's eyes. The monkey chair was equipped with a response button, which was positioned so that the monkey could easily manipulate it. An infrared charge-coupled device camera was firmly attached to the chair by a steel rod in order to monitor eye movements. During the training and recording sessions, the monkey's eye positions were monitored at a time resolution of 33 ms with an eye-monitoring system (Matsuda, 1996). A juice reward was accessible to the monkey through a small spout that was controlled by an electromagnetic valve. A PsyScope system (Carnegie Mellon University, Pittsburgh, PA, USA) controlled the electromagnetic valve and sound signals as well as the timing of the outputs to the cathode ray tube monitor.

### Visual stimuli

**Figure 1:**
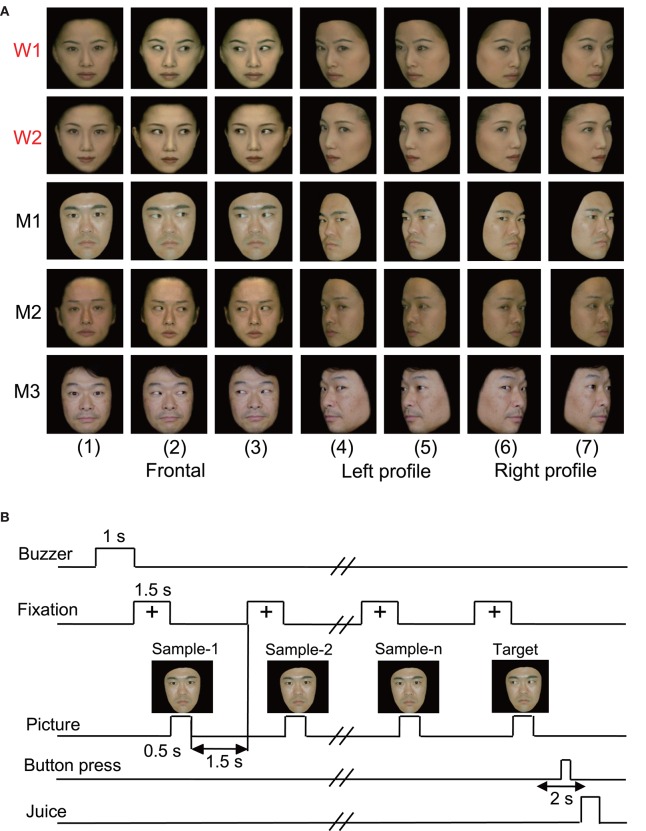
**Visual stimulus set (A)** and task paradigm **(B)** used in the present study. **(A)** The 35 facial photos of five different models, including two females (W1 and W2) and three males (M1, M2, and M3), that were used in the present study. The stimulus set for each model consisted of seven faces with the following different head orientations and gaze directions: (1) frontal view with direct gaze; (2) frontal view with gaze to the right; (3) frontal view with gaze to the left; (4) left profile view with direct gaze; (5) left profile view with indirect gaze; (6) right profile view with direct gaze; and (7) right profile view with indirect gaze. **(B)** The stimulus sequence in the delayed non-matching to sample (DNMS) task in which the stimuli were sequentially presented with a delay between them.

In the original studies, the following five kinds of visual stimuli were used (Nguyen et al., 2013, 2014): human photos, line drawings of faces (cartoon faces), eye-like patterns, face-like patterns that newborn babies orient toward (Johnson et al., 1991), and simple geometric patterns (circle, cross, square, or star). In the present study, we reanalyzed only the responses to the human photos that were reported previously (Nguyen et al., 2013, 2014). Figure 1A shows the stimulus set of photos of human faces that was used in the present study. The facial photos, which were obtained from five human models, consisted of the three following face orientations: straight ahead (frontal face), 30 degrees to the right (profile face), and 30 degrees to the left (profile face). The frontal faces consisted of three gaze directions (directed toward or averted to the left or right of the monkey), and the profile faces comprised two gaze directions (directed toward or averted to the right or left of the monkey). The facial stimuli were 256 digitized color-scale images that were presented on a 0.7-cd/m^2^ black background with their centers at the center of the display. The luminances of these stimuli ranged from 1.36 to 3.66 cd/m^2^, while the luminous intensities (total luminances) ranged from 16.4 to 44.2 mcd. These stimuli were displayed on a cathode ray tube monitor with a resolution of 640 × 480 pixels, and the size of the stimulus area was 5–7 × 5–7°.

### Behavioral tasks

The monkeys were trained to perform a sequential delayed nonmatching-to-sample (DNMS) task that required the discrimination of gaze in the facial photos (Figure 1B). The task was initiated by a buzzer tone. Then, a fixation cross appeared at the center of the display. When the monkeys fixated on the cross for 1.5 s, a sample stimulus was presented for 500 ms (sample phase). The control phase was defined as the 100 ms before the sample phase. When the facial photos were used as sample stimuli, the gaze directions of the stimuli were either directed to or averted from the monkey. Then, after an interval of 1.5 s, the same stimulus appeared again for 500 ms, and this occurred between one and four times (selected randomly for each trial). Finally, a new stimulus with a different gaze direction was presented (target phase). When the target appeared, the monkey was required to press the button within 2 s to receive a juice reward (0.2 mL). When the monkey failed to respond correctly during the target phase or to press the button before the target phase, the trials were aborted, and a 620-Hz buzzer tone was sounded. The intertrial intervals were 15–25 s.

In the DNMS task with facial photos, the monkeys were required to discriminate gaze direction (Nguyen et al., 2013, 2014). In the facial pairs, averted gazes were always paired with directed gazes, and stimulus pairs of gazes averted to the left and the right were not used. Furthermore, the facial stimuli that were presented in the sample phase were the same as in the target phase, apart from gaze direction (i.e., same model and same head orientation). Thus, the monkeys were required to detect a difference in gaze direction (directed vs. averted gaze). The monkeys required about 11 months of training in order to reach a 97% correct-response rate before surgery (Nguyen et al., 2013, 2014). The monkeys' performance during the recording was stable, and no significant difference in reaction time among the facial stimuli was observed (Supplementary Table 1).

### Electrophysiological procedures and data acquisition

The monkeys were trained in the delayed nonmatching-to-sample task for 3 h/day, 5 days/week. After completion of this training period, a head-restraining device, which was a U-shaped plate made of epoxy resin, was attached to the skull under aseptic conditions (Nguyen et al., 2013, 2014). After the monkeys relearned the delayed nonmatching-to-sample task and were correct at least 85% of the time, we commenced recording neuronal activity from each hemisphere in both subjects. A glass-insulated tungsten microelectrode (0.8–1.5 MΩ at 1 kHz) was stereotaxically inserted into the SC and pulvinar vertically to the orbitomeatal plane. The analog signals of the neuronal activities, visual stimulus triggers, juice rewards, button presses, and X-Y eye position coordinates were digitized at a 40-kHz sampling rate and stored in a computer through a multichannel acquisition processor (Plexon Inc., Dallas, TX) system. The digitized neuronal activities were isolated into single units by their waveform components with the Offline Sorter program (Plexon Inc.). The data that were used in the present study were previously reported in Nguyen et al. (2013, 2014), and more details of the procedures can be found in those studies.

### Analysis of the basic characteristics of the SC and pulvinar neurons

We analyzed the activity of single neurons during the 500-ms period after (*post*) the onset of stimulus presentation in the sample phase, but we did not analyze the activity of single neurons in the target phase. Only the stimuli that were presented more than five times in the sample phase were analyzed. The baseline firing rate was defined as the mean firing rate during the 100-ms before stimulus onset (*pre* period). The significance of the excitatory or inhibitory responses to each stimulus were compared between the 100-ms *pre* and 500-ms *post* periods with a Wilcoxon signed-rank test. *P* < 0.05 were considered statistically significant. Furthermore, the 500-ms *post* period was divided into 10 50-ms epochs in order to investigate the temporal changes in the neuronal responses. The mean neuronal firing rate was calculated for each of these epochs. Response magnitude was defined as the mean firing rate in each epoch minus the mean firing rate during the 100-ms *pre* period.

### Population coding by the SC and pulvinar neurons

MDS, which is a method that is used to simplify the analyses of relationships that exist within a complex array of data, constructs a geometric representation of the data in order to determine the degree of the relationship between the stimuli represented by the data matrix (see Young, 1987 for more details). In the present study, the 35 visual stimuli (facial photos) were used to elicit neural activity in the SC and pulvinar neurons.

Data matrices of the neural activity in each epoch of the 112 visually responsive neurons in the SC and the 68 visually responsive neurons in the pulvinar were generated in a 112 × 35 array and a 68 × 35 array, respectively. The Euclidean distances between all of the possible pairs of visual stimuli were calculated with the visual responses in each epoch of the 112 SC neurons or 68 pulvinar neurons. The MDS program (PROXSCAL procedure, SPSS statistical package, version 16; IBM Corporation, Armonk, NY, USA) positioned the visual stimuli in two-dimensional space with the Euclidean distances between the stimuli representing the original relationships (Shepard, 1962; Kruskal, 1964). The clusters of the visual stimuli in the MDS spaces were then analyzed with a discriminant analysis.

In order to investigate the similarities in the representations of the facial stimuli in the MDS spaces between the SC and pulvinar, the Euclidean distances in the SC MDS spaces between the stimuli pairs were compared with those in the pulvinar MDS spaces with Pearson's correlations.

## Results

### Neuronal responses to the facial stimuli

**Figure 2:**
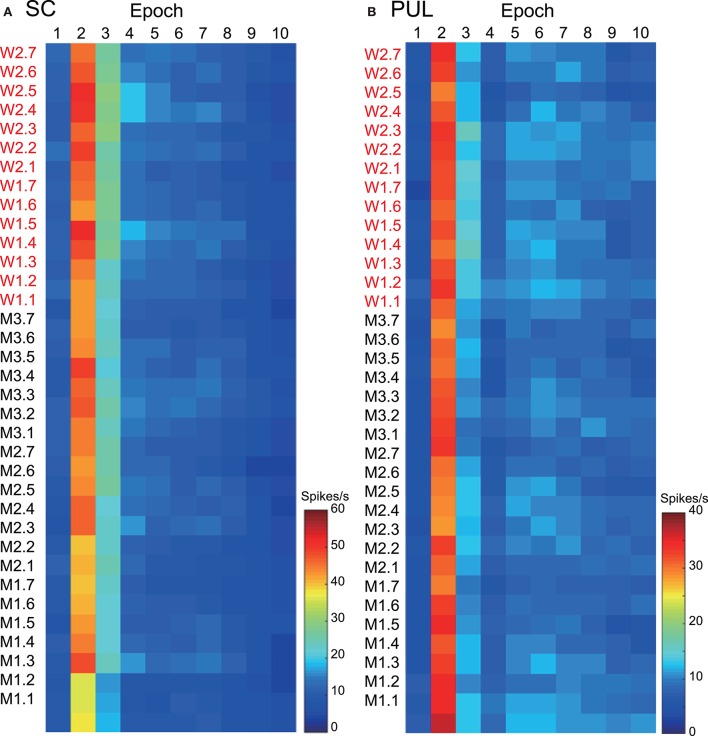
**The temporal changes in the mean response magnitudes of the neurons in the superior colliculus (SC) (A) and pulvinar (PUL) (B) to the facial stimuli across the different epochs**. The labels consisting of two letters (M or W) and the numbers on the left of the graphs refer to the 35 visual stimuli shown in Figure 1A; W1, W2, M1, M2, and M3 before the periods refer to the five models, and the 1 to 7 after the periods refer to the face orientation and gaze direction.

The 112 neurons in the superficial layers of the SC and the 68 neurons in the pulvinar were tested with all of the facial photos (Nguyen et al., 2013, 2014). Only the data from these neurons were used in the MDS analyses. Our previous studies described neuronal responses in the SC and pulvinar in detail (Nguyen et al., 2013, 2014). Figure 2 shows the mean response magnitudes of the 112 SC **(A)** and 68 pulvinar **(B)** neurons to the 35 facial stimuli over 10 epochs. The response magnitudes across the epochs were very similar between the two brain areas; the neurons showed robust responses in epoch 2, and the responses gradually decreased in the following epochs. The pulvinar neurons tended to show more sustained responses compared with the SC neurons. The histological data indicated that most visually responsive SC neurons were located in the superficial layer of the SC (Nguyen et al., 2014), which receives direct visual inputs from the retina (see Introduction), while most pulvinar neurons were distributed in the lateral and medial pulvinar (Nguyen et al., 2013), which receives projections from the SC (see Introduction).

### MDS analyses of the SC and pulvinar neuronal responses

**Figure 3:**
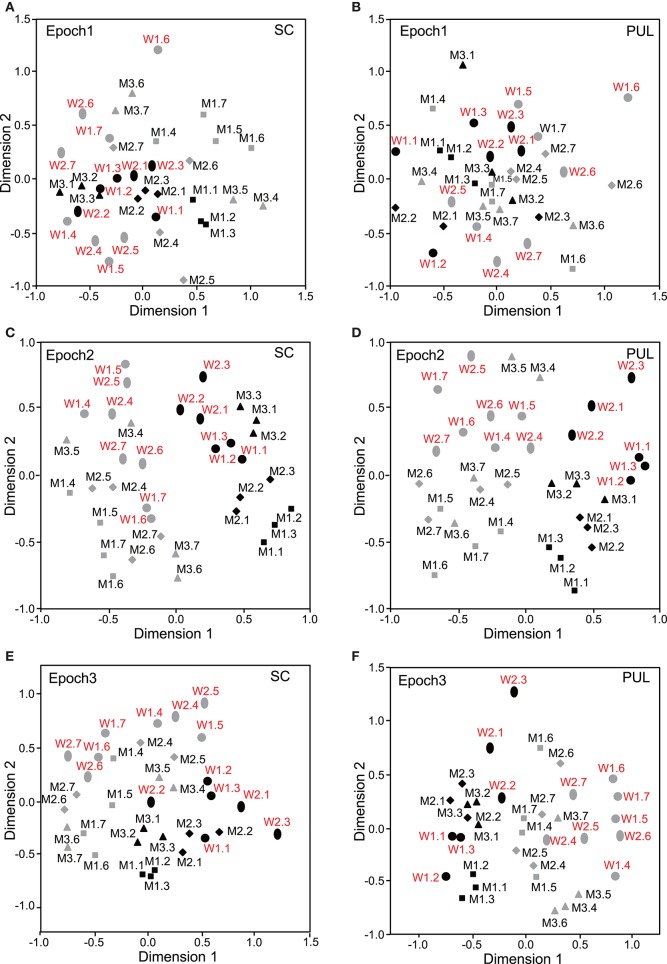
**Distributions of the 35 facial photos in the two-dimensional space resulting from multidimensional scaling (MDS) of the neuronal responses in epochs 1 to 3. (A,C,E)** MDS maps in the SC. **(B,D,F)** MDS maps in the pulvinar. SC, superior colliculus; PUL, pulvinar; black symbols, frontal faces; gray symbols, profile faces; red labels, female photos; black labels, male photos; W1–2, female models; M1–3, male models. See legend for Figure 2 for further explanation.

**Figure 4:**
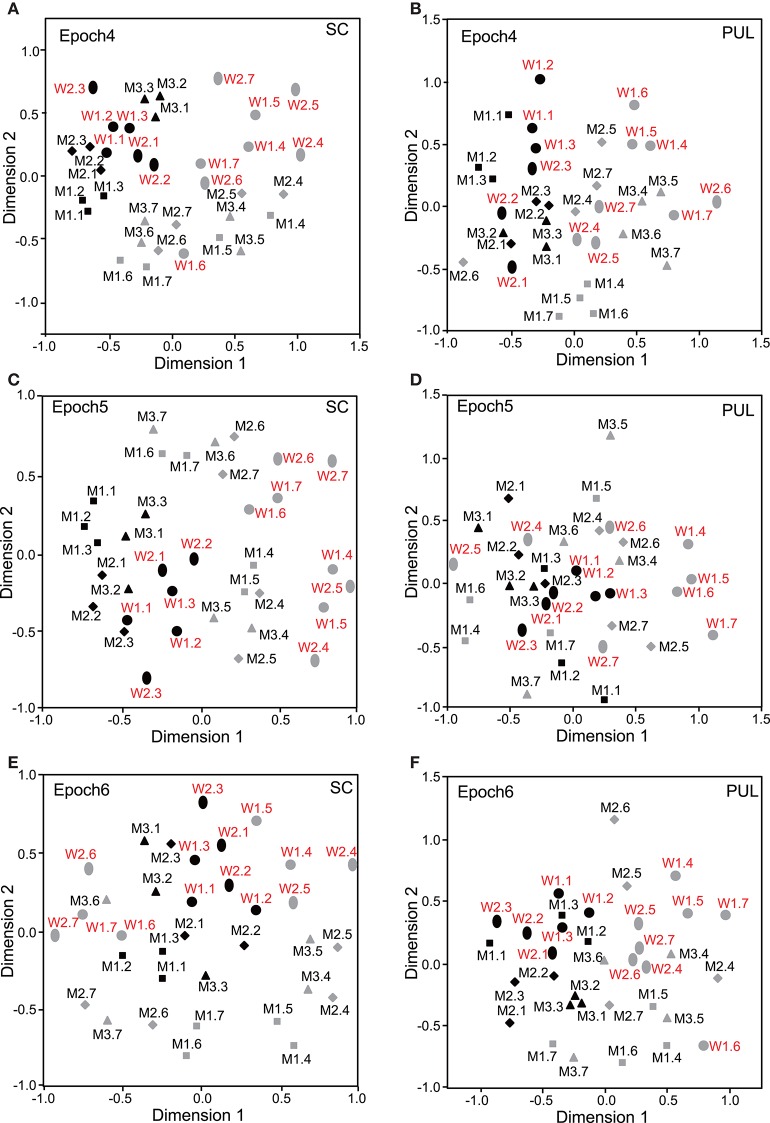
**Distributions of the 35 facial photos in the two-dimensional space resulting from MDS of the neuronal responses in epochs 4–6. (A,C,E)** MDS maps in the SC. **(B,D,F)** MDS maps in the pulvinar. See legends for Figures 2, 3 for further explanation.

**Figure 5:**
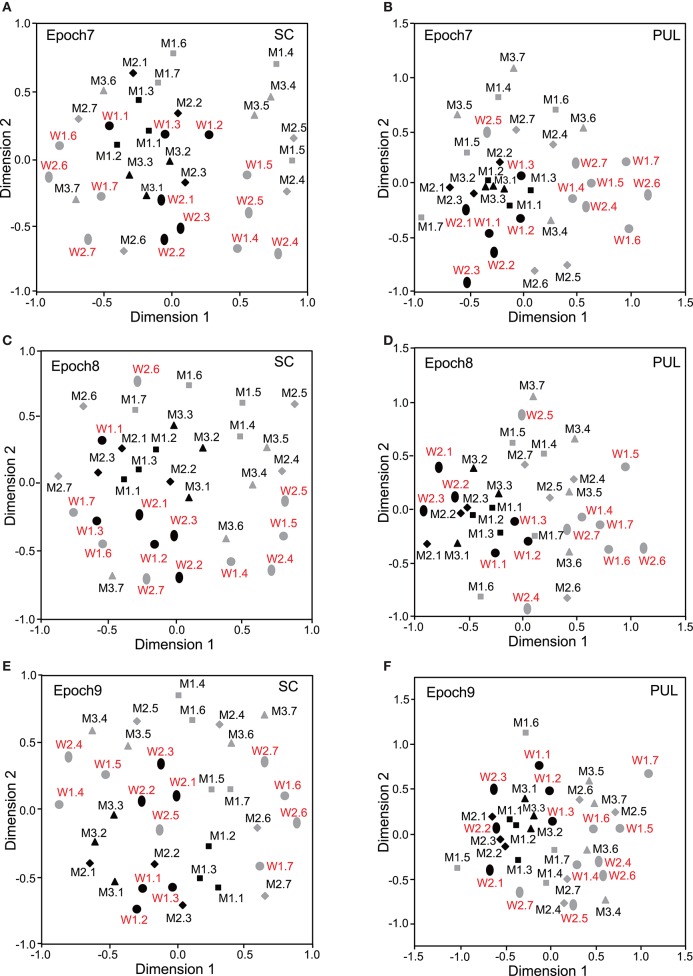
**Distributions of the 35 facial photos in the two-dimensional space resulting from MDS of the neuronal responses in epochs 7–9. (A,C,E)** MDS maps in the SC. **(B,D,F)** MDS maps in the pulvinar. See legends for Figures 2, 3 for further explanation.

The data for the response magnitudes that were recorded from the 112 SC and 68 pulvinar neurons in the 10 epochs were subjected to MDS analyses (Figures 3–**5**). After calculating the stress values and coefficients of determination (r^2^) for up to 4 dimensions, we chose a two-dimensional space (Bieber and Smith, 1986). In the SC, the r^2^ values for epochs 1 to 10 were 0.676, 0.812, 0.768, 0.856, 0.756, 0.815, 0.802, 0.756, 0.671, and 0.689, respectively, for the two-dimensional solutions. In the pulvinar, the r^2^ values for epochs 1 to 10 were 0.821, 0.829, 0.898, 0.828, 0.840, 0.845, 0.836, 0.856, 0.830, and 0.842, respectively, for the two-dimensional solutions. Although some significant clusters were recognized in epochs 1–10 after stimulus onset (see below), no significant cluster was observed in the baseline period (Supplementary Figure 1).

### Classification of face orientation

**Figure 6:**
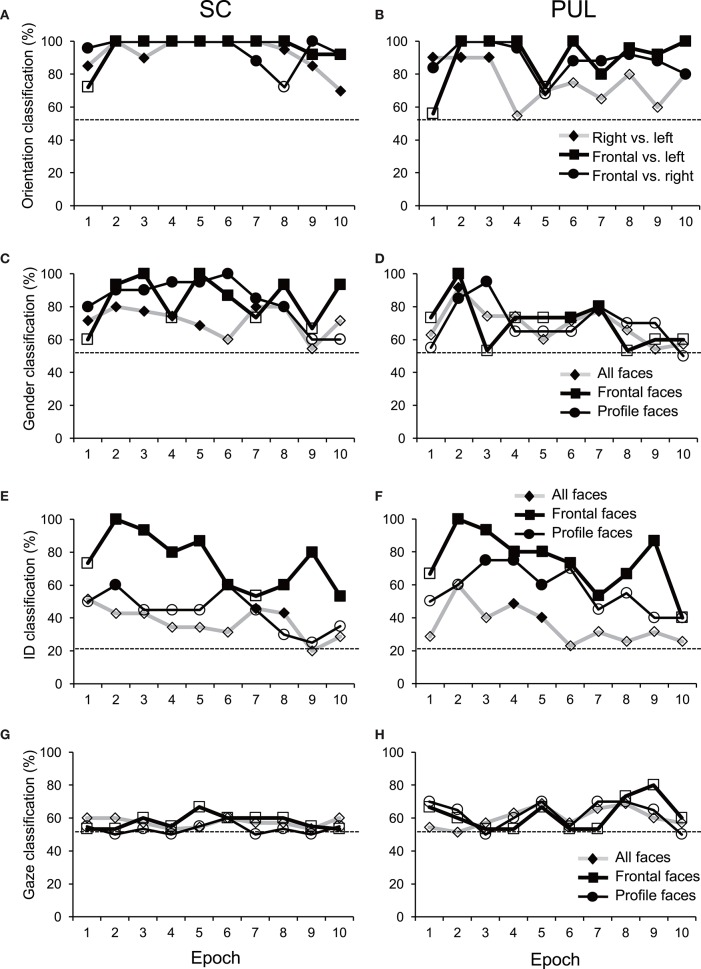
**Discriminant analyses of the facial stimuli in the MDS spaces in the SC (A,C,E,G)** and pulvinar (PUL; **B,D,F,H**). Filled symbols, significant classification (*p* < 0.05); open symbols, non-significant classification (*p* > 0.05). The dotted lines indicate chance levels for classification.

In epoch 1 (Figures 3A,B), the MDS analyses indicated that the 35 facial photos (five models, three orientations, two genders, and two different gaze direction) were intermingled, and no cluster seemed to be recognized in both the SC and pulvinar. However, the frontal faces (black symbols) tended to be located in the center of the MDS space in the SC, while the frontal and left profile faces were mainly located on the left side of the MDS space in the pulvinar. Discriminant analyses of the coordinates of the 35 facial stimuli in the SC indicated that the correct classification rates between the right profile and the left profile and between the right profile and the frontal face were 85 and 96%, respectively, in epoch 1, and these were significant (*p* < 0.05) (**Figure 6A**). However, the correct classification rate between the frontal face and left profile was insignificant (*p* > 0.05). In the pulvinar, the correct classification rates between the right profile and the left profile and between the right profile and the frontal face were 90% and 84%, respectively, which were significant (*p* < 0.05) (**Figure 6B**). However, the classification rate between the frontal and left profile faces was not significant in the pulvinar (*p* > 0.05).

In epochs 2–10 (Figures 3–**5**), both the SC and pulvinar neurons generally showed better categorization of the face orientations compared to that in epoch 1. The frontal faces (black symbols) were always clustered separately, except for the poor classification that was observed in epoch 5 in the pulvinar (Figure 4D). The discriminant analyses (**Figures 6A,B**) indicated that the correct classification ratios between the frontal faces and the left profile faces and between the frontal and the right profile faces were higher than those between the left and the right profiles in most epochs except for epochs 4, 7, and 8 in the SC. In the pulvinar, the correct rates for all of the faces were not significant in epochs 4–10 (*p* > 0.05). These data indicated that in these subcortical structures, classification of face orientation was sensitive to face orientation: the frontal and profile face orientations were more clearly differentiated, while the left and right profiles were differentiated less.

### Classification of gender

The difference between SC and pulvinar neurons was greater in the classification of gender compared with the classification of face orientation. The pulvinar showed clear clustering of gender in epoch 2 (Figure 3D) as the female models (red labels) were located in the upper half of the MDS space, while the male faces (black labels) were located in the lower half of the space. In contrast, the SC showed relatively better gender clustering across all of the epochs except for epoch 9 (Figures 3–5). Discriminant analyses of the SC MDS space indicated that gender was significantly classified for the frontal and profile faces (*p* < 0.05), but the correct rates were lower for all of the faces even though the classification was significant (*p* < 0.05), except for epochs 4, 6, 9, and 10 for all of the faces (Figure 6C). In the pulvinar, the correct rates of gender classification for all of the faces, frontal faces, and profile faces were significant in epoch 2 (*p* < 0.05) and then gradually decreased (Figure 6D).

### Classification of identity

The SC and pulvinar neurons showed very similar trends in the classification of identity of the face models. In the first epoch, clustering of identity was not recognized in either the SC or pulvinar. In epochs 2 and 3, the frontal faces of the same models tended to form clusters, but this tendency was not clear for the profile faces (Figures 3C,F). Later, in epochs 4–9 (Figures 4, 5), the classifications of identity became less clear than they were in epochs 2 and 3, but the identity categorizations of the frontal faces were always better than those of the profile faces. The discriminant analyses (Figures 6E,F) indicated that the correct rates of identity classification were significant for the frontal faces in epochs 2–10 in both the SC and pulvinar (*p* < 0.05). In contrast, the correct rates of classification were lower and sometimes insignificant for the profile faces and all of the faces in both the SC and pulvinar.

### Classification of gaze directions

Gaze discrimination by the SC and pulvinar neurons was very poor. The discriminant analysis (Figures 6G,H) indicated that the classifications between the direct and indirect gazes were not statistically significant in any of the epochs in the SC and pulvinar (*p* > 0.05).

### Similarity of population coding in the SC and pulvinar

**Figure 7:**
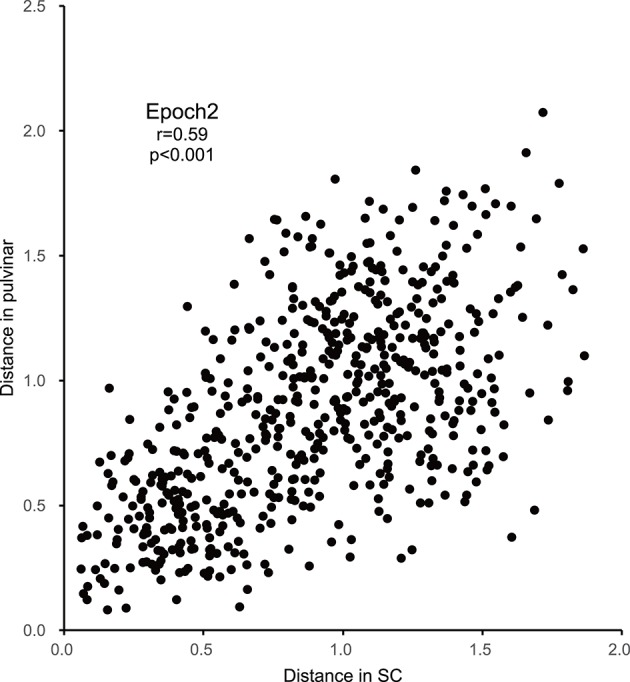
**Correlations between the Euclidean distances between stimulus pairs in the MDS spaces in epoch 2 for the SC and pulvinar (significant positive correlation with *r* = 0.59; *p* < 0.001)**.

In order to examine the similarities of the stimulus representations between the SC and pulvinar, the Euclidean distances between all of the pairs of stimuli in the SC MDS spaces were plotted against those in the pulvinar MDS space in epoch 2 (*n* = 595, Figure 7). The analysis indicated a significant positive correlation between the distances in the SC and pulvinar in epoch 2 (*r* = 0.59, *p* < 0.0001). The remaining epochs, except for epoch 4, also exhibited the following significant positive correlations between the distances in the SC and pulvinar MDS spaces: epoch 1, *r* = 0.148 (*p* = 0.0002); epoch 3, *r* = 0.42 (*p* < 0.0001); epoch 4, *r* = 0.074 (*p* = 0.07); epoch 5, *r* = 0.15 (*p* = 0.002); epoch 6, *r* = 0.16 (*p* < 0.0001); epoch 7, *r* = 0.22 (*p* < 0.0001); epoch 8, *r* = 0.21 (*p* < 0.0001); epoch 9, *r* = 0.10 (*p* = 0.012); and epoch 10, *r* = 0.31 (*p* < 0.0001). These results indicated neuronal populations in the SC and those in pulvinar showed similar stimulus representations.

## Discussion

### Classification of face orientation

In the first 50 ms (epoch 1), both the SC and pulvinar neurons were less sensitive to face orientation, which was consistent with the findings in our previous reports that the latencies of the SC and pulvinar neurons were around 50 ms (Nguyen et al., 2013, 2014). After this initial period, the two brain areas showed clear classifications of face orientation from 50 to 200 ms after stimulus onset (epochs 2–4). In these epochs, the two brain areas similarly discriminated between the frontal and profile faces but did not discriminate between the left and right profiles. For the latencies that were more than 200 ms after stimulus onset (epochs 5–10), although the two brain areas showed diversity in the categorization of face orientation, the frontal faces always formed an isolated cluster, and the boundary between the clusters of the left and right profiles was not clear.

Frontal faces differ from profile faces in a number of characteristics, including peripheral contour and the symmetry and asymmetry of the eyes, mouth, and nose (Valentin et al., 1999). The SC and pulvinar neurons might code for these characteristics. The population coding of face orientation that was observed in the present study was consistent with previous findings in the monkey temporal lobe (Eifuku et al., 2004; Meyers et al., 2015). Furthermore, the lack of discrimination between the left and right profile faces in the SC and pulvinar was similar to previous findings in the monkey posterior superior temporal gyrus in which the neurons responded similarly to the left and right profile faces (De Souza et al., 2005). However, neurons in the anterior superior temporal sulcus discriminated between left and right profile faces (Eifuku et al., 2004; De Souza et al., 2005). These findings suggest that the discrimination between left and right profile faces requires higher cortical visual processing.

Priority of the frontal faces in the categorization of face orientation suggests that these faces may play a more important role in social communication. The posterior temporal cortex has been reported to respond to frontal faces but not profile faces in 5-month-old human infants, while the same cortical region responded to both frontal and profile faces in 8-month-old infants (Nakato et al., 2009). These findings suggest that facial information from the subcortical system might affect the cortical visual system in early infants with premature cortical visual systems. Furthermore, previous behavioral studies have reported that monkeys and humans display behavioral sensitivity to face orientations (Perrett et al., 1990) and that great apes follow the directions of a human experimenter based mainly on the human's face orientation with eye direction also playing a role (Tomasello et al., 2007). These findings suggest that frontal faces with their face orientation directed to the subjects are important social signals regardless of gaze direction. Therefore, the lack of discrimination between the left and right profile faces might be attributed to the social significance of the frontal faces.

However, rapid classification in the MDS might be ascribed to stimulus generalization by repetition of the same stimuli in the DNMS task. In the present study, the monkeys were required to discriminate gaze direction of the facial photos, but not face orientation, identity, nor gender in the DNMS task. Therefore, stimulus generalization process would most affect categorization of gaze direction. However, rapid categorization was observed except gaze direction (see below). These findings suggest that factors other than stimulus generalization (e.g., physical characteristics related to facial orientation as well as facial identity, gender, etc.) might be more important for rapid stimulus categorization in the SC and pulvinar.

### Classification of identity

The classification of identity was significant in epochs 2, 6, and 7 and higher for the frontal faces than for the profile faces in the SC. A similar trend was observed in the pulvinar. Previous human neuropsychological and monkey neurophysiological studies have suggested that the subcortical visual system rapidly processes low-spatial frequency information (i.e., low spatial resolution) (Vuilleumier et al., 2003; Van Le et al., 2013; Nguyen et al., 2013). Newborns with premature cortical visual systems are able to identify faces with low-spatial frequency ranges (de Heering et al., 2008). Psychological studies of adult humans have reported a significant contribution of the low-spatial frequency information of faces to the recognition of face identity (Näsänen, 1999; Schyns and Oliva, 1999). Furthermore, healthy adult human subjects show a monocular (same eye) advantage in the recognition of facial identity when facial stimuli are presented to the same or different eyes, which suggests that the subcortical visual system, which receives monocular information, is involved in the recognition of facial identity (Gabay et al., 2014). The present results provide neurophysiological evidence for a role of the subcortical visual system in the recognition of facial identity.

The present study indicated that frontal, and not profile, faces were categorized well according to individual identity (i.e., view-dependent categorization of facial identity). However, the recognition of facial identity is view-independent in adult humans as well as in infants older than 6 months (Fagan, 1976; Cohen and Strauss, 1979; Pascalis et al., 1998). Furthermore, the population coding of facial identity by cortical face neurons is face orientation (view)-independent (Eifuku et al., 2004; Meyers et al., 2015). In addition, the cortical visual system preferentially responds only to frontal faces in 5-month-old infants, but to both frontal and profile faces in 8-month-old old infants (Nakato et al., 2009). These findings suggest that, in young infants, the cortical facial processing system is more dependent on the subcortical visual system, and consequently more sensitive to frontal faces, which differs from the cortical facial processing system in adults that is view-independent.

### Classification of gender

Gender was also well discriminated in epochs 2 and 3 in the SC and pulvinar in the present study. Consistent with these results, a neurophysiological study has reported that monkeys can behaviorally discriminate the gender of human faces (Afraz et al., 2015). A psychological study reported that facial features, including the brows, eyes, jaws, and chins, carry information about gender difference (Brown and Perrett, 1993). These findings suggest that the population neuron responses in the SC and pulvinar might code for these facial features that are related to gender. It is noted that human infants are sensitive to differences between female and male faces (Quinn et al., 2002; Ramsey et al., 2005). This sensitivity to gender differences seems to be innate, at least in monkey infants (Paukner et al., 2010). These behavioral studies suggest that gender information is processed in the subcortical visual system that plays a major role in infants. Furthermore, a behavioral study has reported that the priming of masked (subliminal) spatially low-pass, but not high-pass, filtered facial images significantly affected the subsequent discrimination of gender (Khalid et al., 2013). Because subliminal low-pass filtered images are thought to be processed in the subcortical visual pathway (Tamietto and de Gelder, 2010), these findings suggest that the subcortical pathway can process gender information (Khalid et al., 2013). In addition, a patient with blindsight was able to discriminate the gender of facial images that were presented to his blind hemifield (Morris et al., 2001). All of the above evidence suggests that gender information is processed in the subcortical system, and the present results provide neurophysiological evidence for the role of the subcortical pathway in gender discrimination.

### Classification of gaze directions

In the present study, the MDS and discriminant analyses indicated that there were no clusters of any specific gaze direction in both the SC and pulvinar in all 10 epochs. A previous neurophysiological study has reported that amygdalar neurons respond more strongly to human photos with a direct gaze than those with averted gazes (Tazumi et al., 2010). Furthermore, this sensitivity to direct gaze was evident only when the neural data were limited during the 100 ms after the stimulus onset, which suggested that this information is derived from the subcortical visual pathway (Tazumi et al., 2010). Analyses of individual neurons showed differential responses to gaze directions in the SC and pulvinar (Nguyen et al., 2013, 2014). These findings suggest that the neuronal responses to gaze directions are not large enough to affect the representation in the MDS space in the SC and pulvinar but are integrated in the amygdala.

### Role of the SC and pulvinar in the subcortical visual pathway

In the present study, the Euclidean distances between all of the pairs of stimuli in the MDS spaces in the SC were significantly correlated with those in the pulvinar in all of the epochs except for epoch 4. In particular, it showed strong correlations in epoch 2 and 3 in which the neurons showed the highest firing rate in response to the stimuli. These results were consistent with those of previous studies, in which the response magnitudes in the SC to the visual stimuli including facial photos were significantly correlated with those in the pulvinar (Nguyen et al., 2014). These findings suggest that the subcortical visual pathway (SC-pulvinar-amygdala) might convey fast and coarse information of visual objects, including faces, to the cortical system (Johnson, 2005; Day-Brown et al., 2010; Tamietto and de Gelder, 2010). However, the categorical information of facial images in the SC and pulvinar may come from feedback signals from the higher cortical structures, where similar information, such as face orientation and facial identity, is processed. The feedback signals from the high-level areas might improve and modify the neuronal responses in the low-level areas (Lamme and Roelfsema, 2000). Consistently, cortical lesions or inactivation substantially changed responses of SC neurons (Wickelgren and Sterling, 1969). However, this was unlikely in the present study at least in the early epochs (epochs 2–3). First, there are important differences in categorization between the cortical and subcortical systems: (1) the identity categorization was view-dependent in the subcortical system, while it is view-independent in the cortical system (Eifuku et al., 2004; Meyers et al., 2015), and (2) the left and right profiles were not discriminated in the subcortical system, while they are well discriminated in the cortical system (Eifuku et al., 2004; Meyers et al., 2015). Second, categorization was evident in the second epoch (50–100 ms after stimulus onset) in the subcortical system (present results), while categorization was evident 100 ms after stimulus presentation in the cortical system (Meyers et al., 2015). These findings suggest that early categorization processing of the facial information in the SC and pulvinar does not depend on feedback signals from the cortical system and is likely to be based on bottom-up information processing in the subcortical pathway.

In the later epochs (epochs 4–10), although correlation of MDS configuration between the SC and pulvinar were significant, the correlation coefficients were much smaller than those in the early epochs (epochs 2–3). This suggests that population coding represents somewhat different stimulus representation between the SC and pulvinar in the later epochs. The different MDS configuration between the SC and pulvinar might reflect different cortical feedback inputs to the SC and pulvinar in the later epochs. However, further studies are required to prove or disprove this idea.

## Conclusions

The population coding by the SC and pulvinar neurons extracted information on facial orientation, identity, and gender. However, categorization of facial identity was insufficient (i.e., view-dependent) in the subcortical system, which was different from the cortical system with view-independent categorization. These findings are consistent with the idea that the subcortical system mainly processes coarse and rapid information on faces. It is noted that categorization of facial orientation, identity, and gender was observed although the monkeys were required to discriminate gaze direction only, but not other features of the facial photos including facial identity, facial orientation, and gender. These findings further suggest that facial information is automatically categorized by innate modules in the subcortical system including the SC and pulvinar in early stages (Vuilleumier, 2000).

## Author contributions

Designing work: HN; Data acquisition: MN, and EH; Data analysis and interpretation: All of the authors.

### Conflict of interest statement

The authors declare that the research was conducted in the absence of any commercial or financial relationships that could be construed as a potential conflict of interest.
