# Supplementary material for: Population Coding of Facial Information in the Monkey Superior Colliculus and Pulvinar
Source: Front Neurosci. 2016 Dec 21;10:583. doi: 10.3389/fnins.2016.00583 (PMC5175414; doi:10.3389/fnins.2016.00583)
Supplement: Supplementary file 1 [file Presentation1.PDF]

Supplementary Material

Population coding of facial information in the monkey superior colliculus and pulvinar

Minh Nui Nguyen, Hiroshi Nishimaru, Jumpei Matsumoto, Quan Van Le, Etsuro Hori, Rafael S. Maior, Carlos Tomaz, Taketoshi Ono, and Hisao Nishijo*

*** Correspondence:** Dr. Hisao Nishijo, nishijo@med.u-toyama.ac.jp

1. **Supplementary Table 1.** Comparison of reaction time to facial stimuli in the SC (A) and pulvinar (B).

A. SC

Model	Frontal Mean ± SD (ms)	Profile Mean ± SD (ms)
W1	296.43 ± 9.24	297.32 ± 8.21
W2	295.34 ± 8.45	295.45 ± 9.36
M1	295.67 ± 8.21	297.24 ± 9.12
M2	296.54 ± 9.11	296.63 ± 7.85
M3	294.85 ± 7.82	295.72 ± 9.26

B. Pulvinar

Model	Frontal Mean ± SD (ms)	Profile Mean ± SD (ms)
W1	295.47 ± 8.21	296.14 ± 8.26
W2	294.76 ± 8.92	295.31 ± 8.36
M1	295.24 ± 8.34	296.28 ± 8.64
M2	295.32 ± 7.65	296.16 ± 7.94
M3	294.68 ± 7.85	295.23 ± 8.22

In both the SC and pulvinar, the data were analyzed by two-way ANOVA, and there were no significant main effects, nor significant interactions (data not shown). W1–2, female models; M1–3, male models. See legend for Fig. 2 for further explanation.

2. Supplementary Figure 1

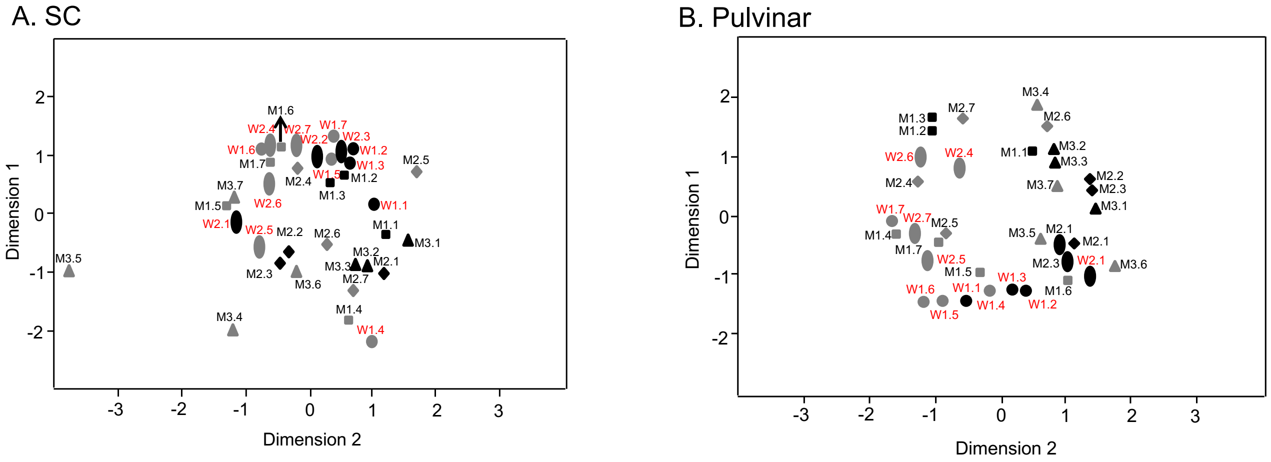

Supplementary Figure 1. Distributions of the neuronal responses in the baseline period to the 35 facial photos in the two-dimensional space resulting from multidimensional scaling (MDS). SC, superior colliculus; black symbols, frontal faces; gray symbols, profile faces; red labels, female photos; black labels, male photos; W1–2, female models; M1–3, male models. See legend for Fig. 2 for further explanation.
